# Early Intervention Guided by the General Movements Examination at Term Corrected Age—Short Term Outcomes

**DOI:** 10.3390/life14040480

**Published:** 2024-04-05

**Authors:** Adrian Ioan Toma, Vlad Dima, Adelina Alexe, Cristina Bojan, Alexandra Floriana Nemeș, Bogdan Florin Gonț, Alexandra Arghirescu, Andreea Ioana Necula, Alina Fieraru, Roxana Stoiciu, Andrada Mirea, Andreea Calomfirescu Avramescu, Al Jashi Isam

**Affiliations:** 1 Life Memorial Hospital, 010719 Bucharest, Romania; 2 Faculty of Medicine, University Titu Maiorescu, 040441 Bucharest, Romania; 3 Neonatology Department Filantropia Clinical Hospital, 011132 Bucharest, Romania; 4 Independent Researcher, 10067 Ploiesti, Romania; 5 Kinetotherapy Department, Pediatric Neurology Alexandru Obregia Hospital, 041914 Bucharest, Romania; 6 Faculty of Medicine, Carol Davila University of Medicine and Pharmacy, 050474 Bucharest, Romania

**Keywords:** general movements, cramped–synchronized, fidgety, early intervention

## Abstract

Background and aim: The early identification of the former premature neonates at risk of neurologic sequelae could lead to early intervention and a better prognosis. This pilot study aimed to investigate whether the General Movement patterns observed at term-equivalent age in former premature infants could serve as predictors for guiding early intervention and improving prognosis. Materials and methods: In a population of 44 premature neonates (mean gestational age 33.59 weeks (+2.43 weeks)) examined at term-equivalent age, 10 neonates with a cramped–synchronized General Movements motor pattern were identified. These neonates were included in an early intervention program consisting of physiotherapy executed both by the therapist and by the parents at home. They were again examined at a corrected age of 12 weeks. The presence or absence of fidgety movements and the MOS-R (motor optimality score revised) was noted. The examinations were performed by certified specialists. Results: Normal fidgety movements and a MOS-R of 20–24 were presented in 9/10 of the former premature infants, with normal foot to foot contact present in 7/10, and normal hand to hand contact present in 5/10. The atypical patterns noted were side to side movements of the head in 5/10, a non-centered head in 9/10, asymmetric tonic neck reflex in 9/10 and jerky movements in 10/10. One patient presented with no fidgety movements and a MOS-R score of 9. Conclusion: Early intervention in our group of patients allowed for an improvement in the neurologic status, demonstrated by the presence of fidgety movements. We suggest that early intervention should be indicated in all premature infants that present with a cramped–synchronized GM pattern during examination at term-equivalent age. However, due to the small sample size, the absence of statistical analysis and a control group, and the limited follow-up period, the conclusions must be approached with caution.

## 1. Introduction

Neuro-motor dysfunction and especially cerebral palsy (CP) represent the main neurologic pathologies of former premature infants, especially NICU graduates [1,2]. Even if metabolic or endocrine disorders can affect also premature infants and lead to neurologic impairments [3], the main causes are represented by lesions of the germinal matrix and/or the white matter [1,2], together with the development of infants’ nervous system ex utero in difficult situations. Even though neonatal intensive care techniques have improved during the last decades, the incidence of CP in preterm infants is still higher than in their full-term counterparts at 2 years corrected age; this was identified by a literature review to be around 7% in low and middle-income countries [4]. In a study published in 2021 about the outcome of the infants admitted to a NICU over 11 years, the incidence of major sequelae (defined as cerebral palsy, general quotient ≤75, severe sensory impairment) was 10.8% (3.8% cerebral palsy) [5]. Another analysis of the literature showed that, even if the incidence of moderate to severe impairment is highest among infants of small gestational ages (22–24 weeks—incidence of 42.2–60.9%), moderate and late preterm infants have less severe disease but still experience adverse neurodevelopmental outcomes; careful follow-up and the early detection of developmental problems is therefore required in all premature infants [6]. 

The care of infants at risk does not end the moment they are discharged from the Neonatal Intensive Care Unit (NICU) [7]. As the paragraph above shows, the neuro-motor consequences of being born preterm could be devastating and lead to an un-favorable neurologic prognosis. This was the reason for the appearance of follow-up programs for NICU graduates that aim to identify infants at risk as soon as possible in order to guide appropriate interventions and to offer the best outcomes possible to patients [8]. The ideal program would identify as early as possible the infants at risk and enable their early inclusion in a rehabilitation program. This early intervention, based more on the risk than a diagnosis, will take advantage of the plasticity of the newborn’s brain, which is maximal during the first months of life [9,10] and will be a strong support for families. It will also serve as a secondary prophylaxis, by correcting the incorrect position resulting from the long NICU stay and stimulating the development of the early motor and neuro-cognitive abilities of the child. 

Even if the meta-analyses did not show that early intervention has a definitive impact on the neuro-motor outcome [11], it is the current practice to try to identify as soon as possible the infants at risk to refer them to the appropriate therapies earlier [12,13]. Indeed, a very recent Cochrane Review (2024) showed that early intervention (defined as an intervention before 12 months of corrected age) probably improves cognitive and motor outcomes during infancy, but not at preschool and school age [11]. 

To guide the early intervention, premature infants at risk should be identified as soon as possible, with one of the key components of the early intervention programs being referral to the rehabilitation specialist as soon as the risk is established [13]. The best tools to identify the risk of cerebral palsy (not to establish a definitive diagnosis) have been identified as the Prechtl Qualitative Assessment of General Movements (sensitivity 98%) and Hammersmith Infant Neurological Examination (HINE) (sensitivity 90%) [12]. In a more recent study on a low-risk premature infant population, a good correlation between GM assessment, HINE and the Griffiths Mental and Development Scale assessment results was identified [14]. 

One of the methods proven to identify infants at risk of neuro-motor impairment as soon as term-equivalent age is met is the General Movements neurologic examination, established by Prechtl [15,16,17,18]. This method represents an observational technique based on the recognition of certain movement patterns—both normal or atypical—that are associated either with normal development or with the risk of neurologic or neurobehavioral impairments [16,17,18,19]. General Movements (GM) represent spontaneously observed movements that are noticed from the intra-uterine life [20], consisting of spontaneous movements of the head, trunk and extremities, and normally occur fluently, are complex, in normal situations have a variable intensity, force and speed, and present with a gradual onset and end [17,18]. They are produced by a subcortical central pattern generator [18,21] and their fluent character is probably caused by the modulation of the pattern by the superior (cortical and subcortical) structures [22,23]. There is an evolution of the GM pattern over the first months of life, with writhing movements present as the normal movement pattern at term; these are replaced by the end of the second month and beginning of the third by fidgety movements (see below the description) as the normal GM pattern [17,18]. They could be observed and recorded until about 5 months of corrected age (20 weeks) when they are replaced by normal voluntary movements [18]. 

Two of the GM patterns are known to be specifically related to the risk of CP or motor impairment: the cramped–synchronized movement pattern detected around term [18,24] and the absence of fidgety movements at 3–4 months corrected age [17,18]. 

The cramped–synchronized movement pattern (CS) is an atypical pattern characterized by the synchronous contraction of all the limb and trunk muscles, with the absence of the normally smooth and fluent character [18]. According to the studies published, the CS pattern is strongly associated with a risk of cerebral palsy (CP) [24]. The earlier the CS pattern appears and the longer this pattern persists, the higher the risk of progression to CP [24].

Fidgety movements, the second of the above-mentioned patterns, appear at approximately 9 weeks of age (corrected age) and consist of movements of small amplitude, moderate speed and variable acceleration in the segments of the body (hands, neck, trunk and feet) in all directions; they are present only during wakefulness in a quiet infant [17]. These movements could represent a fine-tuning of the proprioceptive pathways [19] and are a marker of future normal development [18]. The absence of fidgety movements is a stronger predictor of the future appearance of CP [18]; this absence is associated also with the appearance of other deficits [13,17]. 

Fidgety movements are not the only movement patterns observed in infants. This led to the configuration of a scoring system (Motor Optimality Score Revised—MOS-R) containing the observed motor and postural patterns, the adequacy of the patterns for the age, and the character of the movements, with the presence and character of the fidgety movements added as the most important item [15]. A scoring chart is developed and a thorough description of the principles of scoring the movements is described [15]. The maximum score is 28 and the minimal score is 5 [15]. This score, based also on the optimality concept [25] and categorizing the movement/posture as normal or atypical, has been validated for consistency in the results obtained [15,16]. The score has also been validated for different categories of patients, namely premature infants [26,27], and various other pathologies [28,29]. According to the outcome and validation studies, a score of 25–28 is considered optimal [16,30,31]. A score < 14 is consistently associated with a risk of CP [15]. The MOS-R score has also been used to direct intervention; a score of 25–28 does not require intervention, a score less than 20 requires intervention, and scores between 20 and 24 could require intervention depending on other factors [30,31]. 

The detection of infants at risk allows the patient to be included in an early intervention program. There have been studies of early intervention programs from the NICU [32,33] using movement imitation therapy, with good results. All the meta-analyses stated that there is an important heterogeneity in the interventions used, and that this could influence the results [11,34,35] and make the evaluation of the interventions difficult [35]. So, the question is as follows: does the type of intervention matter? For the interventions beyond the NICU, the three components were as follows: education, parent support and therapeutic child development support components [35]. The early intervention guidelines [13] and studies [34,35] suggest that the therapy should be family-centered, based on creating an enriched and mentally nurturing environment [36], and should be aimed at stimulating the infants’ behavior and at solving the problems of everyday life. Another systematic review and meta-analysis suggested that the interventions should consider including psychosocial support for the mothers and measure the outcomes for both mothers and children [37]. 

Our research aimed to assess whether the initiation of an intervention consisting of physical/kineto-therapy performed by both specialists and parents, in former premature infants identified to have a cramped–synchronized GM pattern at the examination at term-equivalent age, could lead to an improvement in the GM pattern detected at 12 weeks corrected age, i.e., the presence of fidgety movements and a MOS-R score in the optimal to moderate suboptimal range as a marker of an improved neuro-motor outcome.

The presence of fidgety movements was chosen as the outcome measure and marker of efficacy of the intervention because the presence of this GM pattern has a very good predictive value for the absence of CP and motor impairment in former NICU graduates [15,17]. Between 95 and 98% of the infants in which fidgety movements are absent will develop CP [15,17]. As a consequence, normal fidgety movements are a marker of future normal development, so identifying them in an infant at risk is reassuring from the point of view of development. See also the above discussion regarding the anatomical substrate of fidgety movements. Also, as previously stated, MOS-R scores over 24 are associated with normal development. Identifying these features in a former premature infant represents a good prognostic sign for future normal motor development [15,17], which demonstrates the efficacy of the therapy. The fact that fidgety movements are present at a very young age compared with other prognostic features, the simplicity of the assessment and the non-invasive examination technique that does not pose any risks to the examined patient were other factors that supported our choice.

## 2. Materials and Methods

### 2.1. Patients and Intervention

The patients included in this study were former premature infants identified to have a cramped–synchronized general movement pattern at the neurologic examination conducted at 40 weeks corrected age (term equivalent age) in a previously published research [38]. The movement patterns observed were described according to the classical studies in the field [17,18].

The approval of the ethics committee was obtained at the same time as that for the previously published research, and informed consent was obtained from the families. 

The baseline characteristics of the subsample studied can be found in reference [38]. 

The former premature infants identified by this method to be at risk of future motor impairment [24] were referred to intensive physical therapy/kineto-therapy with different specialists located in their area (the patients’ domiciles were situated in different places in Bucharest and throughout the country). The therapists were also trained in the General Movements assessment technique and were aware of the result of the initial evaluation. 

The intervention applied to the patients consisted of physical therapy and positioning. The technique used for intervention was the choice of the therapist and consisted of either Incipient Bobath Therapy [39] or Vojta Therapy [40]. The requirements were as follows:

- The physical therapy should be applied for 12 weeks.
- The intervention of the therapist should be performed at least three times per week.
- The parents should be involved in the therapy of the infant by being taught to execute some of the simple techniques, and they should work with the infant daily.

As a more detailed description of the methods used, during the first two months of therapy, the Vojta Method, Bobath method and the MIT-PB (movement imitation therapy) were used; after 2 months, the Bobath method was used. 

The principle of the Vojta method consists of the activation/use of reflex locomotion to obtain, at least in part, elementary movement methods [41]. The Vojta therapy is performed by applying pressure with a precise direction in well-determined regions of the body and in specific postures that result in movement responses; these are defined always as the same pattern, and are immediately occurring and automated [41]; such stimuli, according to the founders of the method, determine two movement complexes: reflex rolling and reflex creeping [41]. For the patients in the study, two variations of the first complex of movement were used: reflex supine rolling and reflex side-lying rolling. The procedures were performed by the therapist.

The second technique, namely movement imitation therapy [32], was first executed by the therapist and then implemented as a tool to be performed by the parents. The therapist recognizes when an abnormal movement pattern occurs and induces a normal, variable movement pattern in the infant. In particular cases with cramped–synchronized movements, the parent/provider recognizes the appearance of a cramp and induces in that moment a normal, variable movement pattern of the arms and legs. The method is described in reference [32]. Teaching the parents how to perform this technique means that the parents can observe the infant more than the provider and can perform these gestures many times a day, increasing the efficiency.

The third method used was the Bobath method [42]. The concept behind this technique is to continuously evaluate and maximize the benefits of the recovery of the child by actively guiding the infant towards new movements [42]. The therapy is focused on improving the posture, mobility and motor functions of the patient, and the infant is permanently evaluated as new acquisitions occur. During the rehabilitation program, the therapy is focused on eliminating asymmetric postures, head control and the initiation of isolated movements of the arms and wrists. The parents are taught posturing and mobilization gestures as part of the “games” they play with the child. 

### 2.2. Measurements and Outcome

The patients were re-assessed at 12 weeks of corrected age in the follow-up program at Life Memorial Hospital, during the regular follow-up visit. General movement examinations were performed to identify the presence of fidgety movements and the Motor Optimality Score—Revised examination, using a standard form [15]. The following were noted:

- The presence or absence of fidgety movements, as a marker of the risk of future motor impairment.
- The Motor Optimality Score Revised (R).
- The score at different subsets of the MOS-R (observed movement patterns, age-adequate motor repertoire, observed postural patterns, movement character).
- The performance at certain items (normal/atypical). From the observed movement, patterns were chosen for the following items: hand-to-hand contact and foot-to-foot contact. These items were selected for their importance in affirming the age adequacy of the motor repertoire [15] and the fact that they show the status of arms (hand to hand contact) and legs (foot to foot contact). From the subset of observed postural patterns, the following were selected: head-centered and asymmetric tonic neck reflex; these were chosen for their importance in assessing the posture of the head, neck and body [15].

The methodology for assessing the general movement patterns was as follows, according to the previously mentioned requirements [15,17,18]:

- The first examination was performed at 40 weeks corrected age. The baby was undressed to just the pampers and laid on a flat, white surface in a lit, heated room that was free of distractions; they were filmed from above in a vertical plane. At least 5 minutes of film of a time period in which the infant was quiet was analyzed by a trained evaluator [AA]. The presence of a normal movement pattern (writhing movements), a poor repertoire or a cramped–synchronized pattern was noted. The normal pattern—writhing movements—consists of a small to moderate amplitude and a slow to moderate speed, is elliptical in form, and has nice rotations of the trunk and head [17,18]. A poor repertoire pattern is represented by a sequence of successive movements that is monotonous and movements of the part of the body that do not occur in a complex way [17,18] The cramped–synchronized movement pattern (CS) is an atypical pattern characterized by the synchronous contraction of all the limb and trunk muscles, with the absence of the normally smooth and fluent character [18]. The patients that presented with a cramped–synchronized movement pattern were selected for further intervention.
- The second examination was performed at 12 weeks corrected age in the same conditions as above. The video record was also evaluated. The presence of fidgety movements was first noted [15,17,18]. Fidgety movements represent movements of small amplitude, moderate speed and variable acceleration in the segments of the body (hands, neck, trunk and feet) in all the directions; they are present only during wakefulness, in a quiet infant [17]. Then, the MOS-R score was determined using the scoring sheet provided in Reference 15. In the same paper, the different items and the scoring are described. The score was determined also by trained providers [AA and AIT]

Since this was a descriptive study, comparative statistics techniques were not used to assess the effect of the intervention. Just the incidence of fidgety movements and the MOS-R scores for each patient were mentioned. The absence of a comparison group limits the value of the findings and the potential for generalization. 

## 3. Results

Among the population of 44 premature neonates (mean gestational age 33.59 weeks (+2.43 weeks) examined at term-equivalent age, 10 neonates were identified as having a cramped–synchronized General Movements motor pattern. The baseline characteristics of the sample are presented in Table 1. Germinal matrix–intraventricular hemorrhage was classified according to Volpe [43], periventricular leukomalacia according to the classification proposed by de Vries [44] and lenticulostriate vasculopathy according to Sisman et al. [45].

The results of the evaluation at 12 weeks corrected age are shown in Table 2. Fidgety movements were present in 9/10 patients, normal foot-to-foot contact was present in 7/10 patients, and normal hand-to-hand contact was present in 5/10 patients. The atypical patterns noted were side-to-side movements of the head in 5/10 patients, the head not being centered in 9/10 patients, asymmetric tonic neck reflex in 9/10 and jerky movements in 10/10 patients.

**Figure 1:**
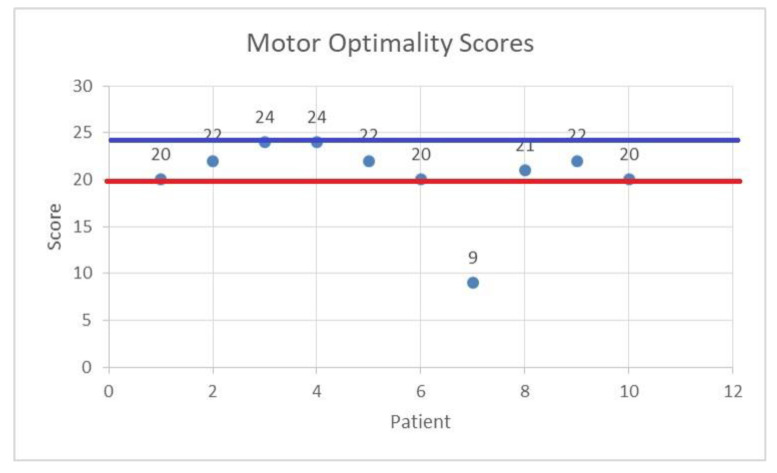
Motor optimality scores for the 10 patients: horizontal axis—patients; vertical axis—MOS-R score. The 2 lines represent cut-off values for the MOS- R score. The red line is the value of 20, below which intervention (in this case kineto-therapy) is mandatory. The blue line represents the value of MOS-R of 24, above which the score is considered normal and no intervention is needed.

Figure 1 presents the results of the MOS-R scores for the 10 patients. The red lines noted are represented by a score of 20, below which intervention is mandatory [15], and a score of 24, considered to be the limit of normal, above which intervention is not usually needed.

As can be observed, all but one of the patients scored above or equal to 20 on the MOS-R.

## 4. Discussion

Our research showed that early intervention in the case of former premature infants with a cramped–synchronized GM pattern could lead to an improved GM examination at 12 weeks of corrected age (presence of fidgety movements). The presence of fidgety movements at that age is a marker of future normal/near normal development, and the absence of CP or a low-grade CP [15] (grades 1 or 2 GMFCS) [46].

There are several issues to be discussed regarding these results. First, according to the medical literature, the natural evolution of a cramped–synchronized GM pattern could be to absent or abnormal fidgety movements, never to normal fidgety movements [17]; so, obtaining a normal fidgety pattern in this sample should be related to the early administration of treatment. Second, studies are showing that early therapy could lead to the modification of the natural evolution of the movement pattern and a better prognosis; in those cases, the interventions were initiated in the NICU [32,33]. The early intervention performed in this sample could be a strong point of the study. The intervention consisted of a combination of Bobath and Vojta therapies. This method has been applied with good results also by another group, working independently from ours [47], though neither the moment of evaluation nor the endpoints were the same. Third, early intervention is proven to act on the quality and organization of fidgety movements even after a short course of therapy; another research group showed that, in the case of infants with mild postural asymmetries, the temporal organization of the fidgety movements was improved after an early motor training procedure [19].

The strong points of this research could be considered to be the early identification of patients at risk using a neurologic exam proven to have a good predictive value [12,13,17] and the use of the same technique in assessing the results of the therapy [13]. Also, the early intervention in this case could be considered a strong point. Even if the literature considers early intervention a program of therapy initiated in the first year of life [35], we considered that acting as quickly as possible could lead to better results by taking advantage of the plasticity of the neonatal and small infants’ central nervous system [9,10].

The main limitations of this study are the small sample size and the lack of a control group. Indeed, the number of patients is small, but the large majority in which de-intervention is efficient could strengthen the value of the results. The study’s small sample size and lack of a control group limit the ability to generalize the findings and firmly establish causality. To address these limitations, we propose conducting additional statistical analyses, such as t-tests or ANCOVA for longitudinal studies, to provide more robust insights. Furthermore, incorporating a control group in future research endeavors is essential for drawing more definitive conclusions regarding the efficacy of early intervention strategies in this population. The absence of a control group could be regarded from the point of view of ethics as follows: we identified in the study a group of patients with a CS pattern that is known to be associated with the risk of cerebral palsy [17,19,24]. Early intervention is mandatory in this situation in our opinion, so we expected this to be efficient. Also, early intervention in patients at risk of cerebral palsy is not considered harmful according to the medical literature [34,35]. Indeed, the meta-analyses did not find that physical therapy had an effect on the outcome of the patients at risk of cerebral palsy, but this could be due to the heterogeneity of the interventions and the outcome measures used [34,35]. The heterogeneity of the interventions could be considered also a weak point of this research. Indeed, different therapeutic methods were used by different therapists, but the results were almost the same. The meta-analyses in the field suggested the same concept, that physical therapy has the same result, not depending on the technique used; the main factors for a good outcome were the involvement of the families and the interventions being oriented towards movements initiated by the child and a nurturing home environment [34,35]. The education and involvement of the families has been the common approach in all the early interventions in this program, and even though we could not draw a statistically significant or evidence-based conclusion regarding this approach, we believe that this involvement of the families made a difference in our early intervention.

The case of the patient who did not present with fidgety movements at 3 months corrected age deserves further discussion. The patient had a gestational age of 30 weeks, had a complicated hospital course and presented with a cystic form of PVL identified by the head ultrasound. Why did we not succeed in this patient when we did in others? The answer resides probably in the severity of the lesions of this patient. Grade III PVL [44] signifies cystic lesions in the periventricular white matter, related to the destruction of all the cells and the motor pathways [48]. In the case of the other children, the CS pattern could have as a substrate the destruction of certain cellular lines (the sub-plate neurons) [22,23,49] and the lesion could be bypassed by the plasticity of the small infants’ brain. This is speculation, and the question could not be answered by this research. As a future research topic, it could be investigated whether early identification and therapy have the same effect in all patients with a cramped–synchronized movement pattern or if the effect is related to the type of lesions reflected by this abnormality of the neurologic examination.

Another issue to be discussed is the results of specific items in the MOS-R scores. As we can see, the worst results were noted in the observed postural pattern sections, with an asymmetric head position and an asymmetric tonic neck reflex noted. There could be several explanations for this: first, the asymmetric tonic neck reflex persists for a long period and can be found in a sub-clinic fashion in older infants and children, as shown by several pediatric neurology schools [50,51]. The asymmetry could also reflect the posture of the premature infant in the NICU, not yet corrected by physical therapy [51].

In the observed movement patterns section, the maturation seems to go in a caudo-cranial direction: normal foot-to-foot contact is present before normal hand-to-hand contact. The normal foot-to-foot contact that was present in 7/10 patients in our study is reassuring, because an association between atypical foot-to-foot contact and future occurrence of CP was described in [15], where it is mentioned that all the patients with normal foot-to-foot contact had present fidgety movements. Hand-to-hand contact, even if not present, does not have a strong association with future neurologic impairment, and still has time to develop in this cohort of patients [15].

The main implications of the findings of our pilot study for clinical practice are the following. The early identification of infants at risk by using the GM evaluation allows for early intervention and the efforts of the follow-up team to be concentrated on the infants at risk. The early evaluation at 12 weeks by the same technique allows the team to validate the effectiveness of the intervention and, at the same time, intensify efforts and change the techniques used in the case of an abnormal movement pattern. So, we achieved the early identification, early intervention and early evaluation of the efficacy in a category of patients in which time has a significant effect on the brain. In conclusion, using this approach, we gained time that can be efficiently used in the efforts to fully treat these infants. 

Maybe the most important point for practice is that we could and should make parents a part of the team and could, in this way, improve the outcomes of the families and infants.

## 5. Conclusions

Our data showed that early intervention in the case of former premature infants identified as having a cramped–synchronized GM pattern at the examination performed at term-equivalent age resulted in the improvement of their neuro-motor status, as shown by the appearance of normal fidgety movements in the vast majority of the patients. The presence of fidgety movements is strongly associated with a low risk of cerebral palsy, so we could anticipate a good neuro-motor prognosis in these cases.

We consider early intervention and the involvement of the families in the management of the infants to be important in obtaining these results.

The limitations of this study are important and worth mentioning: The sample is too small to draw statistically significant conclusions about this fact, and further research on larger samples is needed to confirm these findings; the absence of a control group and the short follow-up period also limit the value of the conclusions. Nevertheless, we recommend early intervention in the case of former premature infants identified as having a cramped–synchronized GM pattern at term-equivalent age and the involvement of the families as partners in performing the rehabilitation procedures on the child.

As previously stated, the many limitations of this research limit the value of the conclusions drawn. We emphasize once more that caution should be used in drawing conclusions based on this research. Indeed, further research in this direction is necessary in order to validate the predictive value of General Movement patterns in guiding early intervention in former premature infants.

## Figures and Tables

**Table 1:** Baseline characteristics of the sample.

Gestational Age	Gender	Birth Weight (g)	Head Circumference (cm)	Head Ultrasound	CPAP (hours on)	MV (days on)	Antibiotics Days	NEC	Days on TPN	GM Pattern	Fidgety Movements	Motor Optimality Score
30	M	980	29	PVL grade3	0	5	7	no	10	CS	absent	9
30	M	1000	29	normal	0	5	14	yes	12	CS	present	20
32	F	900	27	normal	0	0	7	no	8	CS	present	20
32	M	1350	29	normal	24	0	14	no	7	CS	present	20
34	F	1900	31	normal	0	0	5	yes	7	CS	present	21
32	F	1300	29	LSV grade1	0	2	7	no	8	CS	present	22
33	M	1100	28	IVH grade II	0	48	11	no	2	CS	present	22
32	F	1200	27	normal	0	4	26	no	7	CS	present	22
32	F	1100	26	IVH grade I	72	0	10	no	1	CS	present	24
32	F	1100	28	LSV grade 1	24	0	7	no	8	CS	present	24

Legend: M—male; F—female; PVL—periventricular leukomalacia; LSV—lenticulostriate vasculopathy; IVH—germinal matrix/intraventricular hemorrhage; CPAP—continuous positive airway pressure; MV—mechanical ventilation; NEC—necrotizing enterocolitis; TPN—total parenteral nutrition; GM—general movements; CS—cramped–synchronized.

**Table 2:** Results of the general movements evaluation and motor optimality scores at 12 weeks corrected age.

Gestational Age	GM Pattern—40 Weeks Corrected Age	Fidgety Movements	Motor Optimality Score	Observed Motor Patterns (Selected)			Age Adequate Motor Repertoire	Observed Postural Patterns (Selected)			Movement Character	
				Hand to Hand Contact	Foot to Foot Contact	General Score		Head Centered	Asymetric Tonic Neck Reflex	General Score	Type	Score
30	CS	absent	9	no	no	2	2	no	no	2	jerky	2
30	CS	present	20	no	no	2	2	no	no	2	jerky	2
32	CS	present	20	no	yes	2	2	no	no	2	jerky	2
32	CS	present	20	no	no	2	2	no	no	2	jerky	2
34	CS	present	21	no	yes	4	2	no	no	1	jerky	2
32	CS	present	22	yes	yes	4	2	no	no	2	jerky	2
33	CS	present	22	yes	yes	4	2	no	no	2	jerky	2
32	CS	present	22	Yes	yes	4	2	no	no	2	jerky	2
32	CS	present	24	yes	yes	4	4	no	yes	2	jerky	2
32	CS	present	24	yes	yes	4	4	yes	no	2	jerky	2

Legend: GM—general movements; CS—cramped–synchronized.

## Data Availability

The database of the study can be accessed upon request at the address adrian.toma@prof.utm.ro.
